# Bacteriophages in Hip and Knee Periprosthetic Joint Infections: A Promising Tool in the Era of Antibiotic Resistance

**DOI:** 10.3390/medsci14010009

**Published:** 2025-12-25

**Authors:** Filippo Migliorini, Luise Schäfer, Raju Vaishya, Jörg Eschweiler, Francesco Oliva, Arne Driessen, Gennaro Pipino, Nicola Maffulli

**Affiliations:** 1 Department of Trauma and Reconstructive Surgery, University Hospital of Halle, Martin-Luther University Halle-Wittenberg, 06097 Halle (Saale), Germany; joerg.eschweiler@uk-halle.de; 2 Department of Orthopaedic and Trauma Surgery, Academic Hospital of Bolzano (SABES-ASDAA), 39100 Bolzano, Italy; 3 Department of Life Sciences, Health, and Health Professions, Link Campus University, 00165 Rome, Italy; 4 Department of Orthopaedic and Trauma Surgery, Eifelklinik St. Brigida, Kammerbruchstr. 8, 52152 Simmerath, Germany; luiseschaefer83@gmail.com; 5 Department of Orthopaedics and Joint Replacement Surgery, Indraprastha Apollo Hospital, Sarita Vihar, New Delhi 110076, India; raju.vaishya@gmail.com; 6 Department of Trauma and Reconstructive Surgery, BG Klinikum Bergmannstrost Halle GmbH, 06112 Halle (Saale), Germany; 7 Department of Sports Traumatology, Università Telematica San Raffaele, 00166 Rome, Italy; olivafrancesco@hotmail.com; 8 Department of Orthopaedic and Trauma Surgery, Luisenhospital, 52064 Aachen, Germany; arne.driessen@luisenhospital.de; 9 Department of Orthopaedics, Villa Erbosa Hospital, IRCCS Ospedale S. Raffaele, 40119 Milano, Italy; dottgennaropipino@yahoo.it; 10 Department of Trauma and Orthopaedic Surgery, Faculty of Medicine and Psychology, University La Sapienza, 00185 Roma, Italy; n.maffulli@qmul.ac.uk; 11 School of Pharmacy and Bioengineering, Faculty of Medicine, Keele University, Stoke on Trent ST4 7QB, UK; 12 Centre for Sports and Exercise Medicine, Barts and the London School of Medicine and Dentistry, Mile End Hospital, Queen Mary University of London, London E1 4DG, UK

**Keywords:** biofilm, suppressive antibiotic therapy, personalised phage preparation, chronic infection, relapse, *Staphylococcus aureus*, therapy

## Abstract

**Background**: Periprosthetic joint infections (PJIs) of the hip and knee are one of the most severe complications in arthroplasty, often requiring prolonged antibiotic therapy and multiple revision surgeries. The increasing prevalence of multidrug-resistant organisms and biofilm-associated PJIs has renewed interest in bacteriophage therapy as a targeted, adjunctive treatment option in refractory cases. This investigation systematically reviews and discusses the current evidence regarding the application, outcomes, and safety profile of bacteriophage therapy in the management of PJIs. **Methods**: This systematic review was conducted in accordance with the 2020 PRISMA statement. PubMed, Google Scholar, EMBASE, and Web of Science were accessed in August 2025. No time constraints were used for the search. All clinical studies investigating bacteriophage therapy for bacterial PJIs were considered for eligibility. **Results**: A total of 18 clinical studies, comprising 53 patients treated with bacteriophage therapy for PJI, were included. The mean follow-up was approximately 13.6 months. *Staphylococcus aureus* was the most frequent pathogen (18 cases); phage cocktails were used in 33 patients and monophage preparations in 9, all combined with suppressive antibiotic therapy. Persistent or resistant joint pain was reported in only two patients (3.8%), while signs of ongoing infection despite phage therapy were observed in four patients (7.5%). Adverse events following BT were inconsistently reported. **Conclusions**: Bacteriophage therapy shows promise as an adjunctive treatment for hip and knee PJIs, especially in refractory or multidrug-resistant cases. Current evidence is limited and methodologically weak, underscoring the need for well-designed clinical trials to clarify efficacy, safety, and optimal integration into existing orthopaedic infection protocols.

## 1. Introduction

Total hip and knee arthroplasties are among the most effective procedures in orthopaedic surgery, offering substantial improvements in mobility [1,2], pain reduction, and quality of life for patients suffering from advanced degenerative joint diseases [3,4]. With ageing populations, expanding surgical indications, and evolving implant technology, the number of hip and knee replacements continues to rise globally [5,6]. Despite their clinical success, these interventions are not without complications [7,8,9,10,11,12,13]. Periprosthetic joint infection (PJI) remains one of the most serious and challenging complications after total joint arthroplasty, with reported incidence ranging from 0.5% to 2% in primary implant settings and up to 10% in revision settings [14,15,16,17]. The clinical management of PJI typically requires prolonged antimicrobial therapy in combination with surgical intervention [18,19], including debridement and implant retention in selected acute cases or explantation and staged reconstruction in chronic or relapsing infections [20,21]. However, treatment failure rates remain significant, particularly among patients with impaired host defences, compromised soft tissues, or infections caused by biofilm-forming or multidrug-resistant bacteria, such as *Staphylococcus aureus (S. aureus), Pseudomonas aeruginosa (P. aeruginosa), and Enterococcus faecalis (E. faecalis)* [22,23]. Moreover, current antimicrobial strategies are often limited by poor biofilm penetration, systemic toxicity, and increasing antibiotic resistance, all of which contribute to high recurrence rates and a substantial economic and functional burden [24,25,26]. Within this complex therapeutic landscape, bacteriophage therapy (BT) has re-emerged as a promising adjunct or salvage option for the treatment of difficult PJIs involving the hip and knee [27,28]. Bacteriophages, or phages, are viruses that selectively infect and lyse specific bacterial targets while preserving the host microbiota and surrounding tissues [29,30]. Their natural ability to penetrate biofilms and replicate at the site of infection offers a distinct theoretical advantage in the management of PJIs, particularly when standard therapies have failed or are contraindicated [31,32,33,34]. Although the clinical application of phage therapy has historically been restricted to certain regions, such as Eastern Europe, recent advances in microbiology, genomics, and phage purification have made personalised phage preparations increasingly accessible and safer [35,36,37,38,39,40,41]. Case reports and small series have described favourable outcomes following intra-articular, intravenous, or local phage administration in patients with hip and knee PJIs, including those with multiple prior revisions or limited surgical options [42,43,44,45,46,47,48,49,50,51,52,53,54]. However, the evidence remains sparse, heterogeneous, and largely anecdotal, with variations in phage selection, delivery protocols, and outcome definitions. This investigation systematically reviews and discusses the current evidence regarding the application, outcomes, and safety profile of BT in the management of PJIs.

## 2. Methods

### 2.1. Eligibility Criteria

All clinical studies investigating BT for PJIs were considered for eligibility. Articles published in English, German, French, Italian, or Spanish were included. Only studies corresponding to Levels I to IV of evidence, as defined by the Oxford Centre for Evidence-Based Medicine [55], were eligible. Reviews, editorials, opinion papers, or letters were excluded, as were studies involving animal models, in vitro experiments, cadaveric specimens, computational simulations, or biomechanical analyses. Furthermore, studies lacking quantitative outcome data relevant to this analysis were excluded from the final analysis.

### 2.2. Search Strategy

This systematic review was conducted in accordance with the Preferred Reporting Items for Systematic Reviews and Meta-Analyses (PRISMA) 2020 guidelines [56]. This systematic review has not been registered. To guide the search and ensure methodological transparency, a structured framework was established as follows:

- Problem: PJIs;
- Intervention: bacteriophage therapy;
- Outcomes: clinical results and complications.

A comprehensive electronic literature search was conducted on 26 August 2025 using the PubMed, Web of Science, Google Scholar, and Embase databases, with no publication date restrictions. For PubMed, medical subject headings (MeSH) terms were combined with free-text keywords. For Web of Science, EMBASE, and Google Scholar, keyword-based searches adapted to the syntax of each database were used. All records were exported to EndNote (v 20.6; Clarivate Analytics, Philadelphia, PA, USA), where duplicate records were removed both automatically and manually. Grey literature and trial registries (ClinicalTrials.gov, WHO ICTRP) were screened using exact keywords; however, no additional studies were identified. The full database-specific search strings are reported in Table 1.

### 2.3. Selection and Data Collection

Two authors (J.E. and L.S.) conducted a systematic search across the selected databases. Titles were manually screened for thematic relevance, followed by a thorough evaluation of abstracts from potentially eligible publications. When inclusion appeared likely, full texts were obtained and assessed accordingly. The reference lists of all included full-text articles were also systematically reviewed to capture any additional studies not identified during the initial search. Discrepancies between the reviewers were resolved through discussion and, if necessary, adjudicated by a third senior author (J.E.).

### 2.4. Data Items

Two reviewers (J.E. and L.S.) independently performed data extraction. The following data were systematically retrieved: first author and year of publication; journal name; study design; follow-up duration; number of included patients; mean age; sex distribution; type and duration of BT; key clinical outcomes; and treatment-related complications. Infection control was defined as the absence of clinical signs of infection with or without ongoing suppressive antibiotic therapy, eradication as sustained clinical remission combined with negative microbiological findings after treatment completion, and recurrence as the reappearance of clinical and or microbiological evidence of infection during follow-up.

### 2.5. Assessment of the Risk of Bias and Quality of the Recommendations

The risk of bias was assessed in accordance with the Cochrane Handbook for Systematic Reviews of Interventions [57]. Two reviewers (G.P. and L.S.) independently evaluated the included studies. Case reports were appraised using the Joanna Briggs Institute (JBI) Critical Appraisal Checklist for Case Reports [58], which covers eight domains: patient demographics, medical history (as a timeline), clinical presentation, diagnostic workup, treatment, post-treatment condition, adverse events, and clinical takeaways. Each domain was rated as “Yes”, “No”, “Unclear”, or “Not applicable”. No overall score was generated. Case series were assessed using the JBI Checklist for Case Series [59], which comprises 10 domains covering inclusion criteria, diagnostic reliability, participant selection, reporting of demographics and outcomes, and statistical methods. Non-randomised controlled trials (non-RCTs) were evaluated using the ROBINS-I tool [60], which assesses seven domains of bias: confounding, selection, classification of interventions, deviations from intended treatment, missing data, outcome measurement, and selective reporting. ROBINS-I results were visualised using Robvis software (Risk-of-Bias Visualisation web application; Bristol, UK; available at https://www.riskofbias.info, accessed 3 September 2025) [61].

### 2.6. Synthesis Methods

The statistical analysis was performed by the main author (F.M.) using IBM SPSS Statistics (version 25.0; IBM Corp., Armonk, NY, USA). The approach was based on the recommendations of the Cochrane Handbook for Systematic Reviews of Interventions [57]. Descriptive statistics were used to summarise the extracted data. Continuous variables were reported as arithmetic means and standard deviations. Dichotomous variables were presented as absolute frequencies (events/observations).

## 3. Results

### 3.1. Study Selection

**Figure 1:**
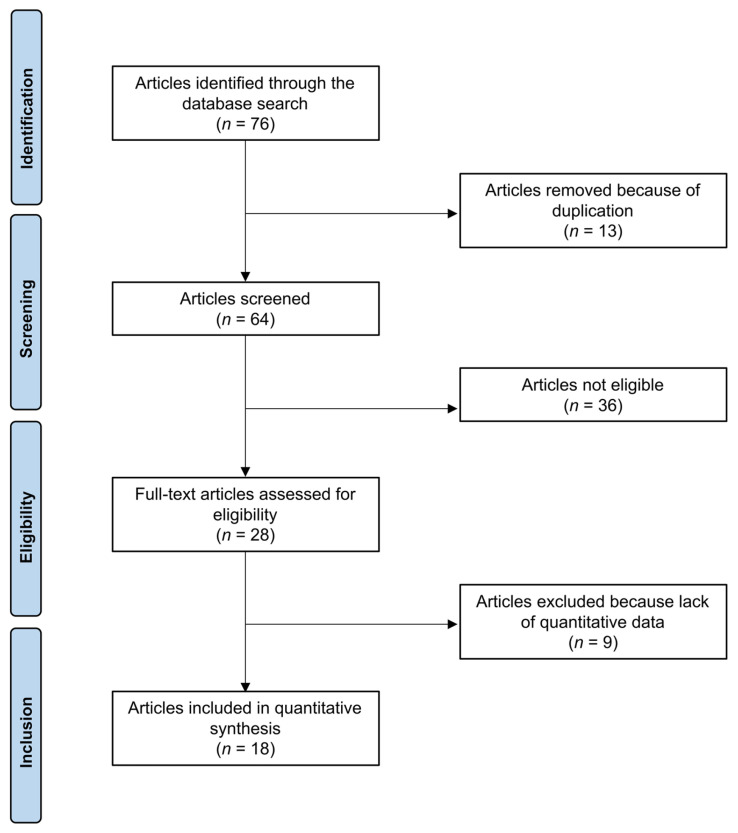
PRISMA flow chart of the literature search.

The literature search resulted in 76 articles concerning the topic of interest. All search results were extracted and checked for relevance. Of these, 13 were discarded because they were duplicates. Following the defined inclusion criteria, abstracts of 64 articles were reviewed, and 36 studies were excluded because they did not fulfil the eligibility criteria. The reasons that led to exclusion were, in detail: study design (*N* = 6), improper level of evidence (*N* = 6), not evaluating PJI of hip or knee (*N* = 13), Insufficient reporting of therapeutic protocol (*N* = 7) and language limitations (*N* = 4). An additional nine articles were excluded because they did not offer quantitative data on the outcomes of interest. Finally, 18 investigations were included in the present analysis. Of them, 17 were case reports or series, and one had a prospective design. The results of the literature search are shown in Figure 1.

### 3.2. Methodological Quality Assessment

The overall methodological quality of the included case reports was high. Most studies provided clear and consistent information regarding patient demographics, clinical presentation, therapeutic procedures, post-treatment course, and reported complications. Follow-up data (Q7) were adequately described in 10 of the 13 case reports, whereas in three reports [42,45,54] follow-up information was either incomplete or not explicitly stated. Despite these minor limitations, the available data in most reports were sufficient to allow critical appraisal and meaningful clinical interpretation. The results of the quality assessment for all case reports are summarised in Table 2.

The five included case series [62,63,64,65,66] were assessed using the JBI Checklist for Case Series, covering ten methodological domains (Q1–Q10). Overall, the methodological quality was moderate, with some variation across studies. Strengths commonly observed included clear inclusion criteria (Q2), transparent reporting of patient demographics (Q3), description of clinical conditions (Q5), and consistent reporting of outcomes (Q7, Q8). Limitations were most frequently noted in the consecutive inclusion of participants (Q1), the level of detail regarding intervention descriptions (Q4), and the completeness of follow-up (Q9). Statistical analyses (Q10) were often not reported or insufficiently described. Only one case series [66] met all ten methodological criteria, reflecting exemplary reporting quality. The remaining series demonstrated generally acceptable methodological standards, but with isolated unclear or missing items. A detailed summary of the quality assessment is presented in Table 3.

**Figure 2:**
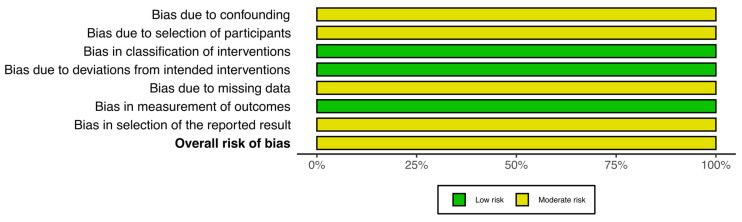
The ROBINS-I of non-RCTs.

Applying the ROBINS-I tool, the study by Fedorov et al. [68] was judged to have an overall moderate risk of bias. Confounding was rated moderate because important baseline factors and potential confounders, such as infection characteristics, causative organisms, and surgical procedures, were documented, but no statistical adjustment was carried out. Selection bias was considered moderate, reflecting the prospective recruitment of the treatment group and use of a historical comparator. The risk related to intervention classification and protocol deviations was low, as treatment allocation was clearly defined and implemented as intended. Missing data introduced a moderate risk, given the incomplete follow-up in some participants. Outcome measurement and reporting were judged to be at low risk of bias. The detailed risk of bias assessment is presented in Figure 2.

### 3.3. Study Characteristics and Results of Individual Studies

Data from 53 patients were included in the present analysis. The mean age was 72.5. ± 11.6 years. The general characteristics, patient characteristics, and main results of the included studies are presented in Table 4. Persistent or resistant joint pain was reported in only two patients (3.8%), while signs of ongoing infection despite phage therapy were observed in 4 patients (7.5%). Adverse events following BT were inconsistently reported. Mild systemic reactions, such as fever or chills, after the first administration were reported in 5 of 53 patients (9.4%). Transient elevations in liver enzymes were documented in 9 patients (17%). Overall, non-specific side effects of any kind were reported in 16 patients, accounting for approximately 30% of the total cohort.

## 4. Discussion

This systematic review highlights the emerging potential of BT as an adjunctive or salvage strategy in the management of PJI. Across the included studies, phages were often administered in complex clinical scenarios in which conventional treatments had failed or were deemed infeasible. While encouraging results were observed for infection control and tolerability, the overall strength of the evidence remains limited. Most of the included studies were case reports or small series, with substantial heterogeneity in patient selection, phage preparation, administration protocols, and outcome reporting. Methodological limitations, including a lack of control groups, incomplete follow-up, and inconsistent documentation of adverse events, restrict the generalisability of the findings and preclude firm conclusions. Nonetheless, the accumulated evidence provides a foundation for further clinical investigation and supports the rationale for developing controlled trials to better define the role of bacteriophages in PJIs.

Beyond the individual case narratives, several consistent cross-study patterns can be identified. Across heterogeneous clinical settings, bacteriophage therapy was almost invariably administered in combination with surgery and prolonged systemic antibiotics, reinforcing its role as an adjunct rather than an independent antimicrobial strategy. A recurrent distinction also emerges between acute PJIs managed with DAIR and chronic scenarios treated with staged revisions or salvage procedures, with phage application shifting from supportive to purely rescue-oriented as biological conditions deteriorate. Moreover, PJIs caused by highly biofilm-adaptive pathogens, particularly staphylococci and Pseudomonas species, appear to represent the most frequent targets for phage intervention. These converging features, despite protocol variability, suggest that the clinical relevance of bacteriophage therapy is shaped less by technical delivery differences and more by the underlying host–pathogen context in which it is deployed. Rather than being viewed as a stand-alone antimicrobial alternative, bacteriophage therapy should currently be interpreted within a rescue-oriented conceptual framework for catastrophic PJIs. The available evidence consistently places phages in scenarios characterised by compromised host biology, biofilm-dominant and multidrug-resistant pathogens, and repeated failure of standard surgical and antibiotic strategies, in which the remaining alternative would otherwise be implant sacrifice or amputation. In this setting, bacteriophages may act as a biologically targeted adjunct capable of reducing local bacterial burden and enhancing antibiotic susceptibility within hostile microenvironments, rather than as a definitive curative therapy [69,70,71,72,73].

The management of PJI involves a range of surgical and non-surgical strategies, adapted to the duration of infection, pathogen characteristics, implant stability, and patient comorbidities [74,75,76,77,78,79]. Debridement, antibiotics, and implant retention (DAIR) are typically indicated for acute PJIs with a symptom duration of less than four weeks [80,81,82,83]. Ideal candidates have a well-fixed implant, intact soft tissues, an identifiable and susceptible pathogen, and no sinus tract or severe immunosuppression [83,84,85,86]. Aslam et al. [42] treated a persistent methicillin-resistant *Staphylococcus aureus* (MRSA) knee PJI with DAIR followed by BT, resulting in complete resolution. A similar outcome was observed by Cesta et al. [44] in a chronic *P. aeruginosa* hip PJI, with sustained eradication during follow-up. Doub et al. [46,62] contributed multiple cases involving *Staphylococcus epidermidis (S. epidermidis) and E. faecalis* PJIs treated with DAIR and BT, with satisfactory infection control. Ferry et al. [47,48,49] included chronic relapsing hip and knee PJIs, frequently involving *S. aureus* or *P. aeruginosa*, which were treated using DAIR and BT with infection resolution [47,48,49]. Ferry et al. [66] reported that one patient underwent amputation one year post-treatment because of prosthesis exposure after a myocardial infarction; the infection itself remained, however, controlled. Patey et al. [65] performed DAIR, closure of several draining fistulae, and BT in a chronic knee PJI. The infection stabilised, but complete eradication was not documented [65]. Several studies performed BT following multiple procedures, ranging from conservative management to revision procedures with implant removal. In most patients, BT represents the last resort. Infection control was achieved in most patients; however, a few remained on suppressive antibiotics, and in some cases, the infection was stabilised rather than eradicated. One-stage revision was reported in several series, including all 23 patients in Fedorov et al. [68], where chronic hip and knee PJIs were managed by implant removal, debridement, and immediate reimplantation. BT was administered intraoperatively via cement and postoperatively through drains [68]. At follow-up, infection control was achieved in most patients, and no recurrences were reported [68]. Patients with more extensive infection or compromised soft-tissue envelopes were managed with a two-stage revision strategy. Ramirez-Sanchez et al. [51] described a persistent methicillin-sensitive *Staphylococcus aureus* (MSSA) knee PJI treated with explantation, cement spacer, intraarticular phage administration, and delayed reimplantation, resulting in infection eradication at 20 months. In Doub et al. [45], a chronic MRSA knee PJI with severe bone loss underwent explantation and static spacer placement; intra-articular and intravenous phages were administered, with negative cultures at reimplantation and no recurrence. Where infection persisted, or local conditions were unfavourable, more complex revision strategies were implemented, ranging from repeated spacer exchanges to a Girdlestone excision arthroplasty. Schoeffel et al. [52] reported a recalcitrant MRSA infection of the hip and knee after multiple failed revisions, managed by sequential single-stage exchange of a hip spacer and knee temporary prosthesis with intra-articular BT, achieving infection eradication at 11 months. Patey et al. [65] described a chronic *P. aeruginosa* knee PJI not suitable for complete revision; partial hardware removal with local phage injection controlled the *P. aeruginosa*, but an Enterococcus infection subsequently emerged. Few patients received BT without any revision surgery. In each instance, surgery was deemed high-risk or technically unfeasible because of severe comorbidities, poor bone and soft-tissue conditions, or multiple prior failed revisions. A patient with chronic *K. pneumoniae* knee PJI with multiple previous revision surgeries was treated using intravenous phages with oral minocycline suppression, with resolution of clinical symptoms and improved function [43]. Neuts et al. [50] reported a chronic relapsing *E. faecalis* hip PJI after failed revisions, managed with oral BT and antibiotics, achieving infection control. A patient with chronic MRSA hip PJI with secondary knee involvement was treated with intravenous and local phages in combination with suppressive antibiotics [54]. The infection was controlled until the patient’s death from unrelated causes [54].

This review has several limitations that must be acknowledged when interpreting the findings. Foremost, the overall low quality of the included studies, with the majority consisting of single-patient case reports and a small number of case series. According to the JBI critical appraisal tools, although most reports adequately described the clinical presentation, intervention, and follow-up, several domains were incompletely addressed, particularly the systematic documentation of adverse events and the clarity of the diagnostic workup. Although a minority of case series demonstrated reasonable methodological standards, only one study fulfilled all ten JBI quality domains, and none included comparator groups or predefined clinical endpoints. The clinical heterogeneity among the reported cases further limits the ability to synthesise results. The spectrum of included patients ranged from those undergoing DAIR procedures for acute infection to others treated after multiple failed revision surgeries, some of whom had severe soft tissue compromise or were considered inoperable. The pathogens involved also varied widely, including methicillin-sensitive and methicillin-resistant *S. aureus, P. aeruginosa, Klebsiella pneumoniae (K. pneumoniae), E. faecalis, and Corynebacterium striatum*, each of which differs in biofilm formation, phage susceptibility, and clinical course [87,88,89]. Moreover, the phage preparations and administration strategies were highly inconsistent. Some patients received intravenously administered purified commercial cocktails, while others were treated with personalised phages selected in vitro and delivered locally, orally, or via drainage systems. The duration and frequency of therapy also varied substantially, and the combination with systemic antibiotics was not uniform. Given these heterogeneities, the reported results cannot be generalised. In addition, the evidence base is likely affected by substantial publication bias. Unsuccessful or inconclusive applications of bacteriophage therapy are less likely to be reported, potentially leading to an overestimation of treatment effectiveness in the available literature. While the overall infection control rate appears encouraging and the safety profile acceptable, these data must be interpreted as preliminary and exploratory rather than definitive. It is also important to note that none of the included studies employed randomisation or blinded assessment of outcomes, and in several cases, treatment success was defined clinically or radiographically without microbiological confirmation. Given the substantial heterogeneity in phage preparations, administration routes, dosing regimens, and concurrent antibiotic strategies, any meaningful subgroup analysis or comparative evaluation across studies was not feasible. These methodological inconsistencies further limited the ability to explore dose–response relationships or to identify protocol-dependent outcome patterns. Future studies should prioritise the design of controlled clinical trials that incorporate microbiological endpoints, standardised definitions of treatment success, and rigorous monitoring of adverse events. Additionally, the development of regulatory pathways for phage preparation, characterisation, and quality control will be essential to support broader clinical application. Despite these limitations, the growing body of clinical experience and the urgent need for alternative strategies for managing refractory PJIs suggest that BT holds considerable promise. Its integration into well-designed translational studies may help define specific indications, optimise administration routes, and clarify its role in combination with surgical and antibiotic treatment. Until such evidence becomes available, current findings should be viewed as hypothesis-generating and supportive of further prospective investigation rather than as a basis for widespread implementation.

## 5. Conclusions

Bacteriophage therapy shows promise as an adjunctive treatment for hip and knee PJIs, especially in refractory or multidrug-resistant cases. Current evidence is limited and methodologically weak, underscoring the need for well-designed clinical trials to clarify efficacy and safety, and to determine the optimal integration into existing orthopaedic infection protocols.

## Figures and Tables

**Table 1:** Medical subject headings (MeSH) and keyword-based searches used as strings for each database.

Database	Search Strategy (MeSH Terms and Keywords)
PubMed	((“Bacteriophages” [MeSH Terms]) OR (“phage therapy”) OR (“bacteriophage therapy”) OR (“phage treatment”)) AND (“Prosthesis-Related Infections” [MeSH Terms]) OR (“Joint Prosthesis Infection”) OR (“Periprosthetic Joint Infection”))
Web of Science	TS = (“bacteriophage therapy” OR “phage therapy” OR “phage treatment”) AND TS = (“periprosthetic joint infection” OR “prosthetic joint infection”)
Google Scholar	“bacteriophage therapy” AND (“periprosthetic joint infection” OR “prosthetic joint infection”)
Embase	(‘bacteriophage therapy’/exp OR ‘phage therapy’ OR ‘phage treatment’) AND (‘joint prosthesis infection’/exp OR ‘orthopedic infection’)

**Table 2:** JBI quality assessment of the included case reports (Q1–Q8). “Y” = Yes; “U” = Unclear.

Author	Q1	Q2	Q3	Q4	Q5	Q6	Q7	Q8
Aslam et al., 2020 [42]	Y	Y	Y	Y	Y	Y	U	Y
Cano et al., 2021 [43]	Y	Y	Y	Y	Y	Y	Y	Y
Cesta et al., 2023 [44]	Y	Y	Y	Y	Y	Y	Y	Y
Doub et al., 2020 [45]	Y	Y	Y	Y	Y	Y	U	Y
Doub et al., 2021 [46]	Y	Y	Y	Y	Y	Y	Y	Y
Ferry et al., 2018 [47]	Y	Y	Y	Y	Y	Y	Y	Y
Ferry et al., 2020 [48]	Y	Y	Y	Y	Y	Y	Y	Y
Ferry et al., 2021 [49]	Y	Y	Y	Y	Y	Y	Y	Y
Neuts et al., 2021 [50]	Y	Y	Y	Y	Y	Y	Y	Y
Ramirez-Sanchez et al., 2021 [51]	Y	Y	Y	Y	Y	Y	Y	Y
Schoeffel et al., 2022 [52]	Y	Y	Y	Y	Y	Y	Y	Y
Tkhilaishvili et al., 2020 [53]	Y	Y	Y	Y	Y	Y	Y	Y
Wahl et al., 2025 [54]	Y	Y	Y	Y	Y	Y	U	Y

(Q1: Was the study question or objective clearly stated? Q2: Was the study population clearly and fully described, including a case definition? Q3: Were the. Cases consecutive? Q4: Were the subjects comparable? Q5: Was the intervention clearly described? Q6: Were the outcome measures clearly defined, valid, reliable, and implemented consistently across all study participants? Q7: Was the length of follow-up well-described?).

**Table 3:** JBI quality assessment of the included case reports (Q1–Q10). Y: Yes; U: Unclear; N: No.

Author	Q1	Q2	Q3	Q4	Q5	Q6	Q7	Q8	Q9	Q10
Doub et al., 2023 [67]	Y	Y	Y	U	U	Y	Y	Y	N	N
Ferry et al., 2020 [66]	Y	Y	Y	Y	Y	Y	Y	Y	Y	Y
Munteanu et al., 2024 [64]	Y	Y	Y	N	Y	Y	Y	Y	Y	N
Patey et al., 2019 [65]	N	Y	Y	N	N	Y	Y	U	U	N

Q1: Were patient’s demographic characteristics clearly described? Q2: Was the patient’s history clearly described and presented as a timeline? Q3: Was the current clinical condition of the patient on presentation clearly described? Q4: Were diagnostic tests or assessment methods and the results clearly described? Q5: Was the intervention(s) or treatment procedures(s) clearly described? Q6: Was the post-intervention clinical condition clearly described? Q7: Were adverse events (harms) or unanticipated events identified and described? Q8: Does the case report provide takeaway lessons?

**Table 4:** Generalities, patient characteristics, and main results of the included studies.

Author, Year	Journal	LoE	Follow-Up (Months)	Patients (n)	Women (n)	Age (Mean)	Joint	DAIR (y/n)	Pathogen	Bacteriophage
Aslam et al., 2020 [42]	*Open Forum Infect Dis*	IV	-	1	1	61.0	Knee	Y	*S. aureus*	AB-SA01, SaGR51ΦK
Cano et al., 2021 [43]	*Clin Infect Dis*	IV	7.8	1	0	62.0	Knee	N	*K. pneumoniae*	Φ2 (KpJH46Φ2)
Cesta et al., 2023 [44]	*Open Forum Infect Dis*	IV	12	1	1	62.0	Hip	Y	*P. aeruginosa*	Pa53
Doub et al., 2020 [45]	*Antibiotics (Basel)*	IV	NR	1	0	72.0	Knee	N	MRSA	SaGR51Φ1
Doub et al., 2021 [46]	*Pharmaceuticals (Basel)*	IV	5	1	1	79.0	Knee	Y	*S. epidermidis*	PM448
Doub et al., 2023 [67]	*Clin Infect Dis*	IV	14	5	NR	NR	Knee, hip	Y	*E. faecalis*, *S. epidermidis*, *S. lugdumensis*, MRSA	EF-1, PM448, Mallokai, SaWIQ0488Φ1, SaGR51Φ1
				4				N		
Fedorov et al., 2023 [68]	*Viruses*	II	12	23	NR	56.0	Hip	N	MSSE, MRSE, MSSA, MRSA	H143, H178, H182, H184
Ferry et al., 2018 [47]	*Open Forum Infect Dis*	IV	18	1	1	80.0	Hip	Y	MSSA	1493, 1815, 1957
Ferry et al., 2020 [48]	*Front Med (Lausanne)*	IV	12	1	0	49.0	Knee	Y	*S. aureus*	PP1493, PP1815
Ferry et al., 2020 [66]	*Front Med (Lausanne)*	IV	30	3	1	82.3	Knee	Y	*S. aureus*	PP1493, PP1815, PP1957
Ferry et al., 2021 [49]	*Front Med (Lausanne)*	IV	12	1	0	88.0	Knee	Y	*P. aeruginosa*	PP1450, PP1777, PP1792
Munteanu et al., 2024 [64]	*Antibiotics (Basel)*	IV	9	1	NR	84.0	Hip	Y	MSSA, *P. aeruginosa*	ISP (Myovirus)
				1	NR	71.0	Hip	N	*K. pneumoniae*, MSSA, *C. striatum*	ISP + SCM (Klebsiella phage)
				1	NR	77.0	Knee	N	*S.epidermidis*	COP-80B (*S. epidermidis*)
Neuts et al., 2021 [50]	*Acta Orthop*	IV	24	1	0	76.0	Hip	N	*E. faecalis*	Pyophages and IntestiPhages in 10 mL vials as an oral suspension
Patey et al., 2019 [65]	*Viruses*	IV	NR	1	1	80.0	Knee	N	*P. aeruginosa*	Commercial broad spectrum multi-bacteriophage suspension
			NR	1	1	72.0	Knee	Y	*Staphylococcus* sp.	Commercial anti-*S. aureus* suspension
Ramirez-Sanchez et al., 2021 [51]	*Viruses*	IV	20	1	1	61.0	Knee	N	MSSA	AB-SA01, J-Sa36, Sa83, Sa87, SaGR51ø1
Schoeffel et al., 2022 [52]	*Pharmaceuticals (Basel)*	IV	11	1	1	64.0	Hip	N	MRSA	SaWIQ0488ø1
Tkhilaishvili et al., 2020 [53]	*Antimicrob Agents Chemother*	IV	10	1	1	80.0	Knee	Y	*P. aeruginosa*	Both *P. aeruginosa* isolates were tested against the phage collection at the George Eliava Institute (Tbilisi, Georgia)
Wahl et al., 2025 [54]	*Front Med (Lausanne)*	IV	NR	1	1	94.0	Hip	N	MRSA	ISP phage (from Queen Astrid Military Hospital)

(PJI: periprosthetic joint infection; MRSA: methicillin-resistant *Staphylococcus aureus*; MSSA: methicillin-sensitive *Staphylococcus aureus*; MSSE: methicillin-sensitive *Staphylococcus epidermidis*; MRSE: methicillin-resistant *Staphylococcus epidermidis*; LoE: level of evidence; NR: not reported; DAIR: debridement, antibiotics, and implant retention; ISP, SCM: phage identifiers as reported in the original studies).

## Data Availability

The original contributions presented in this study are included in the article. Further inquiries can be directed to the corresponding author.
